# “It’s Not Just a System Error”: A Qualitative Study of Nurses’ Perspectives on Medication Safety in Saudi Hospitals

**DOI:** 10.3390/healthcare14131840

**Published:** 2026-06-24

**Authors:** Mukhlid Alshammari

**Affiliations:** Nursing Department, College of Applied Medical Sciences, University of Hafr Albatin, Hafr Albatin 39524, Saudi Arabia; makhlid@uhb.edu.sa; Tel.: +966-583597779

**Keywords:** medication safety, nursing, thematic analysis, EHR, training, workload, incident reporting, Saudi Arabia

## Abstract

**Highlights:**

**What are the main findings?**

- Nurses identified medication safety as a socio-technical process shaped by communication quality, workload, staff competence, and human–technology interaction rather than isolated individual errors.
- Barcode systems and EHRs enhance medication safety, but their effectiveness depends on nurses’ vigilance, adequate training, and supportive teamwork, especially under high-workload conditions.

**What are the implications of the main findings?**

- Improving medication safety requires integrated strategies combining standardized communication (e.g., SBAR, read-back), adequate staffing, continuous training, and non-punitive reporting cultures.
- Healthcare organizations should view technology as a support tool rather than a substitute for clinical judgment, reinforcing human oversight and interprofessional collaboration in medication processes.

**Abstract:**

**Background:** Medication errors remain a major threat to patient safety in acute care settings worldwide and are associated with preventable morbidity, mortality, and increased healthcare costs. Nurses play a critical role in identifying, intercepting, and preventing medication-related harm. However, limited qualitative evidence has explored nurses’ perspectives on medication safety within the Saudi Arabian healthcare context. This study explored nurses’ experiences of medication safety, perceived systemic challenges, and strategies for error prevention in Saudi hospitals. **Methods:** A qualitative descriptive design was employed. Fourteen (*n* = 14) nurses from two major referral hospitals in Saudi Arabia participated in semi-structured face-to-face interviews. Interviews were audio-recorded, transcribed verbatim, and analyzed using Braun and Clarke’s six-phase thematic analysis framework. **Results:** Five overarching themes were identified: (1) Communication gaps; (2) Medication processes; (3) Technology and safety; (4) Workload and staffing; and (5) Staff competence. Participants described how communication failures, staffing pressures, workflow interruptions, and documentation ambiguities compromised medication safety. While barcode systems and EHRs were perceived as valuable safeguards, participants emphasized that their effectiveness depended on staff vigilance, adequate training, and supportive workplace cultures. **Conclusions:** Medication safety is a dynamic socio-technical process shaped by communication, competence, staffing capacity, and human interaction with technology. Improving safety requires integrated organizational strategies that combine workforce investment, structured communication practices, continuous professional education, and non-punitive incident reporting cultures. These findings provide practical insights for healthcare leaders seeking to strengthen medication safety systems in Saudi Arabia and comparable settings.

## 1. Introduction

Medication safety is a major global public health concern, with medication-related incidents accounting for a substantial proportion of preventable patient harm worldwide. The World Health Organization (WHO) reported that medication errors contribute approximately US$42 billion annually in avoidable healthcare costs [1]. In addition, the Organization for Economic Co-operation and Development (OECD) has estimated that 1 in 10 hospitalizations are related to medication harm, and 1 in 5 hospitalized patients experience medication-related harm in member countries [2]. In response to this growing burden, the WHO launched the Global Patient Safety Challenge: Medication Without Harm, calling for coordinated international action to reduce severe, avoidable medication-related harm through safer systems, stronger workforce capability, and improved clinical practices [1].

Medication errors may occur at any stage of the medication-use process, including prescribing, transcribing, dispensing, administering, and monitoring medications [3]. These errors often arise from a combination of human, environmental, technological, and organisational factors rather than isolated individual mistakes alone [3,4]. Common contributors include interruptions during medication rounds, excessive workload, fatigue, inadequate staffing, poor communication, documentation discrepancies, and look-alike or sound-alike medications [4,5]. Because nurses are commonly the final healthcare professionals involved before medicines reach the patient, they play a critical role in identifying discrepancies, intercepting potential errors, monitoring adverse effects, and ensuring safe medication administration [3,4].

Recent patient safety literature increasingly conceptualizes medication safety as a socio-technical process, whereby outcomes are shaped by the interaction between healthcare workers, organizational systems, technologies, workflow design, and team communication [5,6]. Technologies, such as Barcode Medication Administration (BCMA), smart infusion pumps, and Electronic Health Records (EHRs), can reduce certain categories of error and improve traceability [7,8]. However, evidence suggests that technology is most effective when supported by appropriate training, user-friendly design, workflow integration, and ongoing human vigilance. Poor implementation may also create workarounds, alert fatigue, or new forms of risk [7,8].

Within Saudi Arabia, healthcare services have expanded rapidly in recent years in line with Saudi Vision 2030, which prioritizes healthcare quality, digital transformation, and patient safety [9]. Hospitals in Saudi Arabia frequently operate in high-acuity environments with increasingly complex care demands and culturally diverse workforces. Previous studies conducted in Saudi hospitals have reported medication incidents associated with prescribing inaccuracies, administration omissions, communication breakdowns, and staffing pressures [10,11]. In addition, differences in language backgrounds, clinical training, and communication styles within multicultural teams may further influence medication processes and safety behaviors [11].

Although prior studies in Saudi Arabia and internationally have examined medication error prevalence, reporting rates, and staff knowledge, fewer studies have explored the lived experiences of nurses managing medication safety in everyday clinical environments. Qualitative inquiry is particularly valuable because it can illuminate how policies, workload pressures, teamwork, competence, and clinical judgment intersect in real-world practice [12]. Such understanding is necessary to design contextually relevant interventions that move beyond blame-focused approaches and instead strengthen systems of care.

Therefore, the guiding research question for this study was: How do nurses working in Saudi hospitals perceive medication safety, systemic challenges, and strategies for preventing medication errors in clinical practice? Accordingly, the aim of this study was to explore nurses’ experiences and perspectives regarding medication safety, perceived systemic challenges, and strategies for preventing errors in Saudi hospitals.

## 2. Materials and Methods

### 2.1. Study Design

A qualitative descriptive design was used to explore the experiences of 14 nurses regarding medication safety, systemic challenges, and error prevention strategies.

### 2.2. Settings

The study was conducted in two major referral hospitals affiliated with the Saudi Ministry of Health. These institutions provide specialized care for patients with a range of health conditions.

### 2.3. Sampling

A purposive sampling strategy was used to recruit nurses with at least one year of experience in medication administration from two referral hospitals in Saudi Arabia. Participants were invited through flyers placed at nursing stations. Interested nurses contacted the researcher via email and were provided with further study information. Recruitment continued until sufficient depth of data was obtained and no substantially new insights were emerging from interviews.

### 2.4. Participants

A total of fourteen nurses volunteered to participate and completed individual interviews for this study. Among the participants, nine were female and five were male (see Table 1).

### 2.5. Data Collection

Data were collected through semi-structured, face-to-face interviews conducted in private rooms over a period of four months. An interview guide informed by previous literature was used to explore perceptions of medication errors, responses to errors, contributing factors, and prevention strategies (see Table 2). Interviews lasted approximately 30–45 min, were audio-recorded with participant consent, and transcribed verbatim for analysis.

The interviews were conducted by a male researcher with a PhD in Nursing who was working as an academic faculty member at the time of the study. The researcher had previous experience in qualitative research, nursing communication, conducting semi-structured interviews and thematic analysis. This research was prepared and reported following the COREQ checklist for qualitative research reporting.

### 2.6. Data Analysis

**Figure 1:**
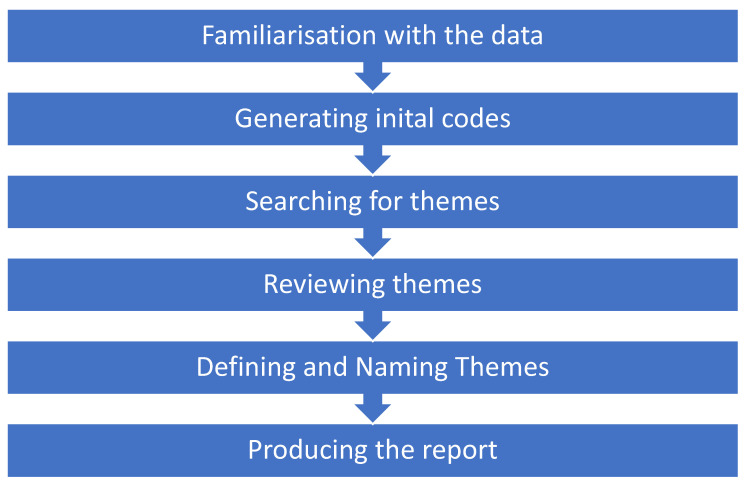
Six stages of thematic analysis by Braun and Clark (2006) [13].

Data were analyzed using Braun and Clarke’s six-phase thematic analysis framework [13], chosen for its flexibility and ability to uncover meaningful patterns in qualitative data [14]. Transcripts were read repeatedly for familiarization, followed by initial coding. Codes were then grouped into categories, sub-themes and broader themes. Themes were reviewed and refined through an iterative process to ensure they accurately reflected participant accounts. A thematic map was developed to illustrate the final thematic structure (see Figure 1).

### 2.7. Trustworthiness

To ensure methodological rigor, the study applied the principles of credibility, dependability, confirmability, and transferability [15,16]. Credibility was supported through repeated engagement with the data and discussion of emerging themes. Dependability was supported through clear documentation of transcription and coding processes. Confirmability was strengthened through transparent links between participant quotations and findings. Transferability was addressed through description of the study context and participant experiences.

### 2.8. Ethical Approval

Ethical approval for this study was granted by the Institutional Review Board of the relevant institution (Approval No. QCH-SRECO 62/2023). All participants provided written informed consent prior to participation. Confidentiality was maintained using pseudonyms, and all study data were securely stored.

## 3. Results

Thematic analysis of the interview transcripts identified five interrelated themes: (1) Communication gaps; (2) Medication processes; (3) Technology and safety; (4) Workload and staffing; and (5) Staff competence (see Table 3). Across themes, participants described medication safety as a shared responsibility shaped by communication, organizational systems, staffing capacity, and professional vigilance.

### 3.1. Communication Gaps

Participants consistently identified communication as central to medication safety. Breakdowns between nurses, doctors, and pharmacy staff were described as common sources of delays, confusion, and preventable risk. These issues were particularly evident during handover, urgent care situations, and when multiple documentation systems were used.

#### 3.1.1. Interprofessional Miscommunication

Participants described how unclear communication between professional groups could result in conflicting orders, delayed treatment, or administration mistakes.

*“Miscommunication tops the list, especially between nurses and pharmacy. I’ve seen orders get garbled like ‘QD’ mistaken for ‘OD’ leading to four doses instead of one.”*

*(Participant 14)*

*“Sometimes the doctor assumes the nurse will catch the change; other times the nurse thinks the doctor’s team has already updated the system. We end up with two versions of the order, neither of which is fully accurate.”*

*(Participant 6)*

Several participants also linked communication failures to high-pressure environments where rapid decisions were required.

*“In high-pressure moments—like a sepsis protocol that needs an antibiotic immediately -this confusion can be dangerous.”*

*(Participant 6)*

*“Even in a code situation that demands emergent dosing, I pause to confirm that the order and medication match before proceeding.”*

*(Participant 9)*

#### 3.1.2. Lack of Feedback Loops

Participants emphasized the importance of structured communication processes such as read-back and Situation, Background, Assessment and Recommendation (SBAR) to reduce ambiguity and confirm verbal changes.

*“We lacked clear ways to confirm information … We’ve started using closed-loop communication. If a doctor changes an order, the nurse repeats it, and the doctor confirms. That simple step has already prevented several near misses.”*

*(Participant 5)*

*“Communication is the backbone of safety. We use SBAR during shift changes and read-back for verbal orders.”*

*(Participant 2)*

These approaches were viewed as practical safeguards rather than optional communication tools.

#### 3.1.3. Unclear Documentation

Participants reported that inconsistent documentation across charts and electronic systems created duplication and uncertainty.

*“We also see transcription errors when the consultant documents in the electronic chart, but the on-call doctor writes a paper order that gets mixed up.”*

*(Participant 4)*

*“Sometimes the medication chart has no allergy alert, or the protocol is unclear, this increases the risk of mistakes … errors emerge from a chain of small lapses rather than a single mistake.”*

*(Participant 1)*

### 3.2. Medication Processes

Participants stressed that structured processes were crucial for medication safety. Practices such as formal double-checks, timely error responses, and consistent documentation were viewed as key components of safe care. These findings illustrate how nurses relied on established systems to verify medications, handle incidents, and uphold standards.

#### 3.2.1. Verification Practices

Nurses described both required and self-initiated verification practices as vital to preventing medication errors. For high-alert medications, protocols included barcode scanning, identity checks, and approval by a second nurse. Some noted that supervisory staff, such as charge nurses, were also involved in verifying high-risk drugs.

*“We scan the identification band and medication barcode; each confirms a match. Every high-alert drug needs two nurses to sign off, and the team leader must verify the order.”*

*(Participant 3)*

*“Never skip a barcode scan or an ID check, no matter how routine the drug seems.”*

*(Participant 6)*

Alongside formal checks, nurses described personal routines such as mental checklists and repeated verbal confirmations, reflecting a dual approach: system-based and individual vigilance.

*“I double-check everything—patient hydration, timing, name, before giving meds, even if I already know the patient.”*

*(Participant 11)*

However, work pressure was reported to compromise these practices.

*“Under pressure, staff may skip checks. During busy times or emergencies, mistakes happen. I once mixed up two patients named Ali.”*

*(Participant 10)*

#### 3.2.2. Standard Protocol Use

Nurses described the use of standardized protocols for medication safety, including storage procedures, infusion settings, and management of high-alert drugs. These routines reduced variability and supported decision-making under pressure. Although digital systems generated alerts for allergies and duplicates, participants emphasized that clinical judgment remained essential.

*“Our unit follows strict protocols… we create a robust safety net.”*

*(Participant 7)*

*“Clear protocols help, especially when things get busy. I know what to check. But even with electronic alerts, you still have to think. The system might flag something incorrectly.”*

*(Participant 12)*

#### 3.2.3. Error Response

When medication errors occurred, nurses described a clear and practiced escalation process that prioritized patient safety through immediate action, assessment, and follow-up. Structured steps such as pausing the procedure, contacting the physician, and documenting the error were commonly reported.

*“The first thing I do is pause the preparation. If I see a dose or timing issue, I contact the doctor and explain.”*

*(Participant 13)*

*“If the wrong fluid is infusing, I stop it immediately, check vitals, then notify the physician and charge nurse.”*

*(Participant 8)*

*“First, I stop any further dosing. Next, I assess the patient’s vital signs and level of consciousness, making sure they’re stable.”*

*(Participant 3)*

However, some participants noted discomfort around reporting, citing fears of judgment or blame.

*“Reporting errors can feel uncomfortable. You worry it might make you look incompetent. The culture isn’t always supportive.”*

*(Participant 12)*

Digital platforms like the Online Variance Reporting (OVR) system were described as important mechanisms for documenting incidents and triggering follow-up investigations.

*“We log the incident in OVR. I complete the form, then pharmacy and leadership follow up.”*

*(Participant 8)*

Participants also valued reflective tools like Root Cause Analysis (RCA) for identifying system flaws and promoting learning.

*“Even as a trainee, I was encouraged to file reports—even minor issues. RCA helps improve policy.”*

*(Participant 5)*

### 3.3. Technology and Safety

Participants saw technology as an important support for medication safety. Tools such as barcode scanners and EHRs were perceived to reduce errors and improved communication. However, nurses emphasized that technology should support, rather than replace, clinical judgment, vigilance, and teamwork.

#### 3.3.1. Barcode and EHR Checks

Participants identified barcode systems and EHRs as important safeguards. These tools enabled real-time checks and were particularly useful during busy shifts.

*“Barcode medication administration reduces up to 50% of wrong-patient/wrong-drug errors. We scan the identification band and medication barcode to confirm identity and match.”*

*(Participant 4)*

*“Once the doctor enters an order, I log in to review it. Our system flags allergies, duplicates, and similar drug names, nothing dispenses until confirmed.”*

*(Participant 13)*

#### 3.3.2. Tech-Aided Workflow

Beyond verification, technology was also seen to improve communication and task coordination. Digital prescribing, automated alerts, and pharmacy integration were described as reducing delays and cognitive burden.

*“When the doctor places an order, I verify it in the system, then message the pharmacy with the urgency. Every order goes straight into their queue.”*

*(Participant 10)*

*“Our system won’t release medication unless scanned by the nurse. It really protects against accidental dosing.”*

*(Participant 6)*

#### 3.3.3. Tech-Limits and Human Role

Despite these benefits, participants cautioned against over-reliance on technology. Errors could still occur due to incorrect data entry, missed alerts, system failures, or labelling issues, highlighting the continued importance of human oversight.

*“Technology alone isn’t enough; we need vigilance and strong processes. We use barcode systems for infusion pumps but still double-check at the bedside.”*

*(Participant 2)*

*“Even with barcode scanning, if a pharmacy label is wrong, it can slip through unless we’re alert.”*

*(Participant 7)*

*“Technology helps, but it’s our team’s vigilance—asking ‘read back’ questions, pausing to verify even routine orders—that truly keeps patients safe.”*

*(Participant 6)*

### 3.4. Workload and Staffing

Fatigue, time pressure, and staffing shortages were commonly identified as risks to medication safety, disrupting protocols and communication. However, participants also described teamwork and peer support as important protective factors.

#### 3.4.1. Fatigue and Overload

Fatigue was frequently described as reducing concentration, judgment, and adherence to safety checks. Long shifts, heavy workloads, and high acuity environments were perceived to increase risk.

*“Stress, if I get overwhelmed, it’s hard to stay focused … most often it’s fatigue, stress, or distraction.”*

*(Participant 9)*

*“Overcrowding, heavy workload, ICU stress, and long shifts all lead to fatigue.”*

*(Participant 12)*

#### 3.4.2. Staffing Issues

Participants noted that safety procedures were more difficult to maintain during emergencies, staff shortages, or patient surges. These pressures could lead to rushed checks, skipped steps, and reduced opportunities for cross-verification.

*“With a 1:6 nurse-to-patient ratio, we feel rushed and may skip checks. Sometimes there are no safeguards, or staff aren’t well trained.”*

*(Participant 11)*

*“When one nurse cares for too many critical patients, it’s hard to double-check every detail.”*

*(Participant 9)*

*“Some nurses only check the first name. In emergencies or busy times, I’ve mixed up patients both named Ali by grabbing the wrong chart.”*

*(Participant 9)*

#### 3.4.3. Peer Support

Despite these challenges, many nurses emphasized the value of informal teamwork in maintaining medication safety. Peer support, task sharing, and a culture of mutual responsibility were described as helping to manage the risks associated with fatigue and understaffing.

*“If one nurse is overwhelmed, others help with checks. We cover each other to prevent tasks from being missed.”*

*(Participant 1)*

*“Our unit values safety. If someone’s unsure, I’ll say, ‘Let’s review the order together.’”*

*(Participant 5)*

### 3.5. Staff Competence

Participants emphasized that ongoing learning, training, and peer support were essential to medication safety. Competence was described as involving both technical skill and confidence developed through experience, mentoring, and reflection.

#### 3.5.1. Training and Orientation

Many nurses attributed their competence to orientation programs and ongoing training. These processes were seen as helping to build safe routines and promote consistency, particularly in settings with high staff turnover or diverse staffing.

*“I’ve been here since orientation and always review protocol updates. That’s helped me avoid mistakes. New nurses should complete supervised probation and annual competencies.”*

*(Participant 8)*

#### 3.5.2. Learning from Errors

Beyond formal training, participants described learning through reflective processes such as Root Cause Analysis and debriefing. These approaches were seen as strengthening practice and supporting a learning culture.

*“We do RCA with all stakeholders to map out why the error happened. Even as a trainee, I was encouraged to report any medication issue so we could learn and improve.”*

*(Participant 5)*

*“Embrace the incident reporting system as a tool for learning, not punishment.”*

*(Participant 6)*

#### 3.5.3. Informal Mentoring

Informal mentoring from senior nurses was described as particularly valuable for new, redeployed, or international staff. Participants explained that peer guidance helped reinforce formal learning and improved confidence.

*“If we’re unsure about a medication, we ask senior staff. New or international nurses often struggle with local systems and drug names, which can cause confusion.”*

*(Participant 7)*

#### 3.5.4. Confidence and Vigilance

Competence was also associated with confidence to act under pressure, combined with sustained vigilance and recognition of the importance of teamwork.

*“I’m confident. We follow safety goals, protocols, barcode checks, pharmacy labeling, and clear communication to catch most issues.”*

*(Participant 14)*

*“My job is vigilance from prescription to pump. Tech helps, but it’s our constant checking that prevents harm. Safety is a team effort.”*

*(Participant 5)*

*“Medication safety is never finished. It’s an ongoing process of learning, system improvement and teamwork.”*

*(Participant 2)*

## 4. Discussion

This study examined nurses’ views on medication safety in Saudi hospitals. The findings demonstrate that medication safety is a dynamic socio-technical process shaped by communication practices, organizational systems, staffing capacity, digital tools, and workforce competence rather than individual performance alone. The five themes identified in this study—communication gaps, medication processes, technology and safety, workload and staffing, and staff competence—reflect the complex realities of medication administration in contemporary hospital practice.

### 4.1. Communication and Coordination

Consistent with international literature, participants identified poor communication between nurses, pharmacists, and physicians as a major contributor to medication errors [2,4]. Breakdowns during handover, verbal order changes, and uncertainty regarding who was responsible for updating medication records were repeatedly described as high-risk points in the medication pathway. These findings align with the World Health Organization’s emphasis on safety communication as a core component of medication harm reduction strategies [1,2].

Participants also valued structured communication approaches such as read-back and SBAR. Current evidence suggests that standardized communication tools improve escalation of concerns, reduce ambiguity, and strengthen interdisciplinary teamwork, particularly in acute care settings [2,11].

A context-specific finding in this study was the coexistence of paper-based and electronic medication systems. Participants described how parallel documentation processes created duplication, outdated orders, and uncertainty regarding the most current prescription. Similar issues have been reported in healthcare settings undergoing digital transition, where partial implementation of electronic systems may create new workflow risks if not fully integrated [7,17].

### 4.2. Medication Processes and Safety Behaviors

Participants described formal verification processes, double-checking, barcode scanning, and escalation pathways as essential safeguards against medication errors. These findings reinforce international patient safety recommendations that medication administration should involve multiple layers of verification, especially for high-alert medicines [1,18].

However, participants also acknowledged that these safety processes were harder to maintain during emergencies, interruptions, or periods of high workload. This is consistent with more recent literature demonstrating that interruptions, competing priorities, and time pressure reduce adherence to medication safety protocols and increase the risk of administration error [4,5].

### 4.3. Technology as Support Rather than Replacement

Technology was generally viewed positively, particularly barcode scanning systems and EHRs, which participants believed improved patient identification, allergy checking, and communication with pharmacy teams. This reflects contemporary evidence showing that barcode medication administration systems can reduce wrong-patient, wrong-dose, and wrong-drug events when appropriately implemented [7,8].

At the same time, participants consistently stressed that technology should support, rather than replace, clinical judgment. They described risks including incorrect data entry, inaccurate labels, missed alerts, and over-reliance on automated prompts. This finding is strongly aligned with current WHO and human-factors guidance, which emphasizes that digital safety tools are most effective when supported by usability design, workflow integration, training, and ongoing professional oversight [1,2,6].

### 4.4. Workload, Fatigue, and Staffing Capacity

Fatigue, cognitive overload, and high nurse-to-patient ratios were repeatedly described as threats to safe medication administration. Participants linked understaffing with rushed checks, reduced concentration, and fewer opportunities for cross-verification. These findings are strongly supported by recent evidence demonstrating associations between inadequate nurse staffing, increased workload, and higher rates of medication administration errors [5].

In high-acuity settings such as emergency departments and intensive care units, these pressures may be amplified by urgent decision-making and frequent interruptions. The OECD has similarly identified medication-related harm as a major systems issue requiring investment in workforce capability, safer staffing models, and real-time organizational learning [2].

Despite these pressures, participants also described teamwork and informal peer support as important protective factors. Task sharing, colleague checking, and collaborative problem-solving were perceived to reduce risk during busy shifts, highlighting that safety is both an individual and collective responsibility.

### 4.5. Staff Competence, Training and Learning Culture

Participants emphasized that orientation programs, continuing education, peer mentoring, and reflective learning processes were central to medication safety. Competence was described not only as technical knowledge, but also as confidence, situational awareness, and willingness to seek assistance when uncertain. This supports current evidence that continuous professional development and supportive supervision improve medication safety capability, particularly among newly employed or transitional staff [5,19].

### 4.6. Contributions and Novelty of the Study

Although themes such as workload pressures, communication failures, and double-checking practices have been reported internationally, this study contributes context-specific insight from Saudi hospitals. In particular, the findings illustrate how medication safety is shaped within multicultural nursing teams, evolving digital systems, and large referral-hospital environments managing high patient acuity.

The study also highlights challenges associated with parallel paper and electronic documentation systems during ongoing health-sector transformation. Given Saudi Arabia’s continuing investment in healthcare reform under Saudi Vision 2030, these findings offer timely evidence regarding frontline medication safety challenges during organizational change [9].

### 4.7. Implications for Clinical Practice and Policy

Several practical implications arise from this study. First, hospitals should strengthen structured communication processes, including SBAR, read-back, and clearer accountability for medication order updates. Second, workforce planning should address the impact of staffing ratios, fatigue, and workload on medication safety. Third, onboarding and continuing education programs should support both local and international nurses to navigate medication safety systems safely. Fourth, digital medication systems should be fully integrated and regularly reviewed to minimize duplicate documentation, usability problems, and alert fatigue. Fifth, healthcare leaders should promote non-punitive reporting cultures that encourage learning from near misses and adverse events.

At a policy level, these recommendations are consistent with WHO’s Medication Without Harm challenge, which calls for coordinated action across medicines systems, healthcare professionals, patients, and organizations to reduce severe avoidable medication-related harm worldwide [1,18].

### 4.8. Limitations

This study has several limitations. The findings were drawn from a relatively small sample of nurses from two hospitals, which may limit transferability to other settings. As data were based on self-reported interviews, participants may have unintentionally underreported personal errors or described practices in socially desirable ways. In addition, the cross-sectional design captured perceptions at one point in time and may not reflect changes in practice over time. Nevertheless, recurring patterns across participants strengthen confidence in the consistency of the findings.

### 4.9. Future Research

Future studies could examine medication safety across a larger number of hospitals and regions within Saudi Arabia, including private and rural facilities. Mixed-methods or observational studies may also help compare reported practices with actual medication workflows. Intervention studies evaluating communication training, staffing models, onboarding programs for multicultural workforces, and digital safety systems would further strengthen the evidence base for medication error prevention.

## 5. Conclusions

This study shows that medication safety in Saudi hospitals depends on nurses’ vigilance, teamwork, communication, and clinical judgment, as well as formal procedures and technology. Despite existing safeguards, staffing pressures, communication gaps, and documentation inconsistencies continue to create risks in medication administration. Improving safety requires integrated strategies that strengthen structured communication, staff training, supportive reporting cultures, staffing capacity, and effective use of digital systems. Medication safety should therefore be understood as a shared, ongoing responsibility requiring both system-level solutions and sustained professional vigilance.

## Figures and Tables

**Table 1:** Participants’ demographic data.

Participant No	Gender	Age	Years of Experience	Qualification
Participant 1	Female	42	6 Years	Bachelor’s degree
Participant 2	Male	39	4 Years	Bachelor’s degree
Participant 3	Female	41	5 Years	Bachelor’s degree
Participant 4	Male	29	2 Years	Associate degree
Participant 5	Female	36	5 Years	Bachelor’s degree
Participant 6	Male	39	6 Years	Bachelor’s degree
Participant 7	Female	45	8 Years	Associate degree
Participant 8	Male	47	16 Years	Bachelor’s degree
Participant 9	Female	44	10 Years	Associate degree
Participant 10	Female	41	9 Years	Master’s degree
Participant 11	Male	40	13 Years	Bachelor’s degree
Participant 12	Female	49	20 Years	Master’s degree
Participant 13	Female	46	4 Years	Bachelor’s degree
Participant 14	Female	41	5 Years	Bachelor’s degree

**Table 2:** Semi-structured interview guide.

1. Describe how medication is prescribed during a shift? 2. What would you consider as an error or omission during the administration of medicines to inpatients? 3. What do you do when there is a medication error? 4. In your opinion, what factors may be related to the occurrence of errors? 5. How can medication errors be prevented? 6. Do you see yourself capable for preventing and solving medication error problems? How?

**Table 3:** Themes and Sub-Themes.

Themes	Sub-Themes
Communication gaps	Interprofessional Miscommunication
	Lack of Feedback Loops
	Unclear Documentation
Medication processes	Double-Check Practices
	Standard Protocol Use
	Error Response
Technology & safety	Barcode & EHR Checks
	Tech-Aided Workflow
	Tech Limits & Human Role
Workload & staffing	Fatigue & Overload
	Staffing Issues
	Peer Support
Staff competence	Training & Orientation
	Learning from Errors
	Informal Mentoring
	Confidence & Vigilance

## Data Availability

The datasets generated and/or analyzed during the current study are not publicly available because they contain information that could potentially compromise participant confidentiality.

## Abbreviations

The following abbreviations are used in this manuscript:

BCMA	Barcode Medication Administration
EHRs	Electronic Health Records
ICUs	Intensive Care Units
OD	Once Daily
SBAR	Situation, Background, Assessment, and Recommendation
OVR	Online Variance Reporting
RCA	Root Cause Analysis
